# Vaccination against Epstein–Barr Latent Membrane Protein 1 Protects against an Epstein–Barr Virus-Associated B Cell Model of Lymphoma

**DOI:** 10.3390/biology12070983

**Published:** 2023-07-11

**Authors:** Wesley I. Soo Hoo, Kaylie Higa, Alison A. McCormick

**Affiliations:** College of Pharmacy, Touro University California, 1310 Club Drive, Mare Island, Vallejo, CA 94592, USA

**Keywords:** Epstein–Barr virus, vaccine, Tobacco Mosaic virus, 38C13 mouse B cell

## Abstract

**Simple Summary:**

The Epstein–Barr virus is a human herpesvirus that infects the majority of the human population. Initially, infection can cause mild symptoms in infants and children, but can also cause short-lived infectious mononucleosis in teenage and young adults. But, in a small minority of older patients, persistent expression of virus antigens can cause B cell transformation to a cancer. Patients with Epstein–Barr virus-positive B cell lymphoma are often more resistant to treatment with standard chemotherapy. One difficulty in developing new methods of treatment is that Epstein–Barr only infects human cells, and there are no mouse B cell models of the disease. Our goal was to develop a mouse model of disease, in order to study the potential for effective vaccination against an Epstein–Barr virus antigen. We were successful in generating a mouse B cell line expressing latent membrane protein 1, and could show that vaccination protected mice against B cell tumors that expressed latent membrane protein 1. This system will allow us to further study the potential for vaccination in patients with cancer, to help the immune system recognize and eliminate tumor cells.

**Abstract:**

In this study, we demonstrate that expression of viral latent membrane protein 1 (LMP1) in a mouse B cell line renders the animals responsive to protection from a 38C13-LMP1 tumor challenge with a novel vaccine. The Epstein–Barr virus (EBV) preferentially infects circulating B lymphocytes, has oncogenic potential, and is associated with a wide variety of B cell lymphomas. EBV is ectotrophic to human cells, and currently there are no B cell animal models of EBV-associated lymphoma that can be used to investigate vaccine immunotherapy. Since most EBV-infected human tumor cells express latent membrane protein 1 (LMP1) on their surface, this viral antigen was tested as a potential target for an anticancer vaccine in a mouse model. Here, we describe a new mouse model of LMP1-expressing B cell lymphoma produced with plasmid transduction of 38C13 into mouse B cells. The expression of LMP-1 was confirmed with a western blot analysis and immunocytochemistry. We then designed a novel LMP1 vaccine, by fusing viral antigen LMP1 surface loop epitopes to the surface of a viral antigen carrier, the Tobacco Mosaic virus (TMV). Vaccinated mice produced high titer antibodies against the TMV-LMP1 vaccine; however, cellular responses were at the baseline, as measured with IFNγ ELISpot. Despite this, the vaccine showed significant protection from a 38C13-LMP1 tumor challenge. To provide additional immune targets, we compared TMV-LMP1 peptide immunization with DNA immunization with the full-length LMP1 gene. Anti-LMP1 antibodies were significantly higher in TMV-LMP1-vaccinated mice compared to the DNA-immunized mice, but, as predicted, DNA-vaccinated mice had improved cellular responses using IFNγ ELISpot. Surprisingly, the TMV-LMP1 vaccine provided protection from a 38C13-LMP1 tumor challenge, while the DNA vaccine did not. Thus, we demonstrated that LMP1 expression in a mouse B cell line is responsive to antibody immunotherapy that may be applied to EBV-associated disease.

## 1. Introduction

The Epstein–Barr virus (EBV) is a gamma human herpesvirus affecting up to 90% of the adult population globally, and preferentially infects B lymphocytes and epithelial cells [1]. Primary infection is often asymptomatic, and may occur during early childhood with the potential to manifest as infectious mononucleosis during adolescence. In most cases after primary infection, the virus remains latent, leading to persistence in memory B cells [2,3,4]. Due to a lifetime persistence in memory B cells, and initiation of latency protein expression that drives B cell transformation, EBV is a known causative agent in the development of B cell malignancies [3]. First discovered in Burkitt’s lymphoma, an aggressive, fast-growing B cell cancer, EBV infection has since been shown to be associated with various B cell lymphomas, nasopharyngeal carcinoma (NPC), and gastric carcinoma (GC) [1,2], and linked to over 300,000 new cases of cancer and over 200,000 cancer deaths worldwide in 2020 [5], and these numbers are expected to grow. Additional recent research has shown that EBV may also be implicated in autoimmune diseases such as multiple sclerosis [6]. Due to the high prevalence of EBV infection and its association with lymphoproliferative disorders, treatment and prevention strategies of EBV-associated lymphomas remain a vital area of research.

EBV has three latency programs, which are marked by selected viral gene expression patterns without EBV virion production [3]. In EBV-associated B cell lymphomas, transformed cells will initially express a total of 11 different latent viral genes. Surface expressed latent membrane proteins LMP1 and LMP2A [4,7] correspond with latency III and II, and are associated with several lymphoproliferative disorders including Hodgkin’s lymphoma, but are also found in diffuse large B cell lymphoma (DLBCL), post-transplant and HIV-related lymphomas, and nasopharyngeal carcinoma (NPC) [2,3,7,8]. The association of EBV latency expression in Hodgkin’s lymphoma ranges from 30–50%, and in post-transplant and HIV-related lymphomas, it ranges from 90–100% [9]. Studies have shown that expression of LMP1 and LMP2A significantly affects the prognosis of lymphoproliferative disorders, and in some cases, can lead to an increased risk of a negative disease outcome [10,11,12,13]. Although both LMP1 and LMP2 are expressed in most EBV-associated cancers, LMP1 is the main driver of tumor progression and tumor escape from immune surveillance, because LMP1 acts as the viral equivalent of CD40 [14]. LMP1 induces the expression of antiapoptotic proteins in EBV-infected cells, impairs immune cell differentiation, and drives B cell expression of growth-promoting cytokines and chemokines [4], which contributes to its enhanced immunosuppressive function, leading to unimpaired cell proliferation [15,16,17,18]. LMP1 is a cell-surface antigen, and, like other B cell immunotherapy targets such as CD20, LMP1 may be an effective target for antibody-mediated tumor immunotherapy [7,19].

There are several challenges to develop an in vivo model for studying potential vaccine strategies in mice. Since EBV only infects human cells, human EBV-transformed lymphocytes such as B-lymphoblastoid cell lines (B-LCLs) have historically been used as a model of EBV-associated lymphoma [20,21]. However, using human B-LCLs as a model of EBV-associated lymphoma necessitates the use of immune-deficient mice to avoid rejection of the human cell lines. The development of humanized mouse models that replicate a human immune system has allowed researchers to study the progression of an EBV-associated lymphoproliferative disorder in NOD/SCID/IL-2Rγ^null^ (NOG) mice implanted with human hematopoietic stem cells [21,22]. However, humanized mouse models of EBV-associated lymphoma still cannot fully replicate a human immune system and retain certain limitations including xenogeneic graft-versus-host disease and lack of MHC-oriented thymus education of T cells, and thus inappropriate hosts for direct immunization and vaccine development studies [23,24]. These limitations highlight the need to develop mouse B cell lines that express EBV latent viral proteins. Considering LMP1 as a potential immunotherapeutic target, native LMP1 is characterized by a large cytoplasmic domain, multiple looping transmembrane sections, and three small extracellular loops [25,26]. Mouse B cell lines that express LMP proteins on the cell surface would allow direct investigation of vaccine efficacy in immune-competent mice, more effectively mimicking an in vivo immune response profile.

Determining the immunogenic epitopes of LMP1 is critical for vaccine development in order to adequately stimulate an immune response. However, the short length of the three extracellular surface loop segments renders them too weakly immunogenic to induce an antibody or cytotoxic T cell response [27]. To overcome this disadvantage, Delbende et al. demonstrated that fusing two loop segments conjugated to a keyhole limpet hemocyanin carrier induced a partially protective humoral immune response in mice after a challenge with LMP1-transformed human embryonic kidney (HEK) 293 cells [25]. In a separate study, Lin et al. used plasmid transduction to produce an epithelial mouse tumor cell line (TC-1) that stably expressed LMP1, which was used to demonstrate DNA plasmid-mediated antitumor vaccine effects in vivo [28]. Although these studies have shown the potential for LMP1 antigen delivery and protective immunity, neither have used a mouse B cell line for vaccine studies. Since the context of antigen presentation is critical to mimic human B cell infection, the choice of cell type for the challenge tumor line may significantly influence important characteristics of the function of LMP1 in vivo, as well as the effectiveness of an anti-LMP1 immunotherapy.

Our present study had two goals: (1) develop a true LMP1-expressing B cell lymphoma mouse model of disease, using stable transformation of the well-characterized mouse B cell line 38C13, and (2) compare the antitumor effects after vaccination with an LMP conjugated peptide epitope vaccine against the viral LMP1 antigen, or a full-length LMP1 plasmid DNA vaccine, in order to establish the mechanism of protection. An LMP1 peptide epitope was fused to a Tobacco Mosaic virus (TMV) carrier, to improve immunogenicity by increasing peptide uptake and activation by antigen-presenting cells [29,30,31], with the goal to improve survival above that observed in the original Delbende experiments, where survival was improved, but all mice died from the tumor challenge [25]. Vaccination with plasmid DNA encoding the full-length LMP1 sequence is more likely to stimulate T cell activation through intracellular antigen presentation, as well as to potentially stimulate humoral immunity to additional epitopes. We compared the potentially immunotherapeutic protection of both types of vaccines in a 38C13 LMP1-expressing mouse B cell tumor model, with the goal to promote survival in a mouse B cell model of EBV-associated lymphoma.

## 2. Materials and Methods

### 2.1. Plasmid Construction and Cell Transfection

The pcDNA3.1 (ThermoFisher, Waltham, MA, USA) mammalian expression vector was selected for transient and stable expression of the full-length LMP1 gene sequence (GenBank: M20868.1), under the control of a CMV promoter. A polymerase chain reaction was used to generate LMP1 inserts, encoding amino acids 1–386, with a C-terminal 6 HIS tag, and confirmed with sequencing. Lipofectamine 2000 (Invitrogen, Waltham, MA, USA) transfection of a pc3.1-LMP1 plasmid into Henrietta Lacks cells (HeLa; American Type Culture Collection (ATCC, Manassas, VA, USA) CRM-CCL2) was initially performed to confirm that the gene insert expressed a full-length LMP1 protein. HeLa cells were grown as per ATCC recommended culture conditions, in minimal essential media (MEM) supplemented with glutamine, 1% penicillin–streptomycin, and 10% fetal calf serum (FCS), in 5% CO_2_ at 37 °C. Cells were plated at a density of 1 × 10^5^ cells/mL for transfection according to the manufacturer protocol. Briefly, 2 µg of DNA was mixed with 2 μL of Lipofectamine in 50 µL of Opti-MEM™ reduced serum media (Gibco/ThermoFisher, Waltham, MA, USA), which was then added to each well of 10^6^ HeLa cells in 0.5 mL of Opti-MEM™, and incubated at 37 °C with 5% CO_2_ for 6 h. The medium from each well was replaced with a fresh antibiotic-free Dulbecco’s Modified Eagle Medium (DMEM), and cells were returned to incubate at 37 °C with 5% CO_2_ overnight. Transfection efficiency was estimated using a Western blot of HeLa cell lysate after extraction with 200 µL of a radioimmunoprecipitation assay (RIPA) buffer. Of that extract, 40 µL was analyzed with SDS-PAGE (BioRad, Hercules, CA, USA) using a dual-color molecular weight marker (BioRad, Figure S1).

### 2.2. Western Blot and Immunofluorescence

Raji cells, a human EBV-infected B cell line (ATCC, CCL-86), were used as a positive control for LMP1 native expression. Raji cells were maintained in Roswell Park Memorial Institute media (RPMI-1640) with GlutaMAX (Gibco), and supplemented with 10% FCS (Atlanta Biologicals). To confirm the integrated plasmids were capable of driving protein expression in transfected cells, as well as in the monoclonal stable cell lines, lysates of 2 × 10^6^ cells were prepared using 200 µL of a RIPA buffer according to the manufacturer’s direction (Invitrogen). Lysates of native Raji cells were prepared as a positive control. In total, 40 µL of protein extracts was separated with SDS-PAGE. Following the transfer of proteins to PVDF membranes, a Western blot analysis was performed in 50 mM of Tris (pH, 7.5), 150 mM of NaCl, 1 mM of EDTA, 0.05% Tween 20, 2.5% milk powder (BioRad, Blotting Grade Blocker), and 2.5% BSA (Sigma, St. Louis, MO, USA; Bovine Serum Albumin), using either rabbit anti-HIS (Genscript A00174-40) or a mouse anti-LMP1 monoclonal antibody (Millipore-Sigma MABF2248) at 1:1000 dilution. Membranes were incubated at room temperature overnight, washed 3 times in 10 mM of Tris (pH, 7.5), 130 mM of NaCl, 1 mM of EDTA, and 0.1% Tween 20 for 15 min followed by incubation for 1 h with an appropriate HRP conjugated secondary antibody at 1:5000 (Southern Biotech, Birmingham, AL, USA) in block. Blots were washed, and then developed with TMB, an HRP substrate (Surmodics, Eden Prairie, MN, USA; Figure S2).

Immunofluorescence imaging of LMP1 expression was carried out after transfection of HeLa cells, as described above. To detect expression of LMP1, cells were washed with PBS and fixed to a 12-well plate for 10 min at room temperature in 3.7% Cytofix/Cytoperm (BD Biosciences, San Jose, CA, USA) in a blocking solution consisting of 2% Probumin diagnostic grade BSA (Millipore, Burlington, MA, USA) in PBS. Cells were treated with a blocking solution for 5 min to prevent non-specific binding. The primary antibody (10 µg/mL mouse anti-LMP1) was diluted in the blocking solution, and the cells were incubated for 1 h at 4 °C. A mouse IgG standard (Pierce/ThermoFisher Scientific) was used as a primary antibody control. Cells were washed 3 times for 5 min each with the blocking solution, then incubated for 30 min at 4 °C in the dark with 2 µg/mL of a fluorescent conjugated secondary antibody (Goat anti-Mouse IgG Alexa Fluor 488 (Invitrogen)) in block. Cells were washed 3 times for 5 min each with the blocking solution, and the staining was visualized using a Nikon Eclipse Ti-S inverted microscope. Images were captured at 100× magnification with a QICAM Fast 1394 Digital Camera using NIS-Elements BR 4.11.00 software (Nikon, Tokyo, Japan) with 10 ms of exposure for bright-field images and 3 s of exposure for fluorescent images.

### 2.3. 38C13 B Cell Transfection and Transformation

We tested various ratios of 1 or 2 µg of GFP DNA (PSc2-eGFP; Clonetech/Takara Bio, San Jose, CA, USA) and 1 or 2 µL of Lipofectamine 2000 (Invitrogen) against 1 × 10^6^ and 2 × 10^6^ 38C13 cells in a 12-well plate, as suggested by the manufacturer instructions. After 24 h, cells were examined for eGFP expression using fluorescence microscopy and each well was scored for GFP-positive cells. Unfortunately, using the ratios suggested for Lipofectamine transfection, no GFP-positive cells were observed. A second test was used to establish if higher DNA and Lipofectamine conditions would give positive results. In total, 1, 2, and 5 µg of DNA were then tested with 2, 5, and 10 µL of Lipofectamine. With 5 µg of DNA and 5 µL of Lipofectamine, only 1 positive GFP-expressing cell was observed per million 38C13 cells 24 h after transfection with fluorescence microscopy. Using 5 µg of DNA and 2 µL of Lipofectamine, three positive GFP-expressing cells were observed. Although of a low efficiency, the 5 µg/2 µL DNA to Lipofectamine ratio was considered optimal, and this ratio was used to transfect 38C13 cells with full-length pc3.1 LMP1 plasmid DNA.

To develop our model EBV LMP1-expressing B cell lymphoma cell line, we transfected 38C13 cells with LMP1 plasmid DNA, and generated stable tumor cell lines with G418 selection (Geneticin, InvivoGen, San Diego, CA, USA). 38C13 cells were transfected with LMP1 plasmid DNA and Lipofectamine 2000, as determined with optimization using a GFP plasmid, in quadruplicate in a 6-well plate, selected for 1 week with 500 µg/mL of G418, then re-plated for clonal selection into 96-well culture plates in 100 µL, at ~25–50 cells/10 mL, and grown for 4 weeks. After 4 weeks, single clones (n = 4) were isolated, expanded, and then passaged twice a week for 5 weeks in RPMI media. Cells were initially screened for LMP1 expression with an anti-HIS Western blot, and then confirmed with an anti-LMP Western blot as described above.

### 2.4. TMV-Peptide and Plasmid DNA Vaccine Preparation

To prepare the LMP1 vaccine, a TMV-LMP1 peptide fusion vaccine was made, similar to that used in a study conducted by Delbend et al. [25] but with an improved carrier for antigen uptake. TMV with an N-terminal surface-reactive lysine (TMV 1295.10; [31,32]) was prepared from infected plants as previously described [32], and then activated with a 10-fold molar excess of sulfo-LC-SMCC, a heterobifunctional crosslinker (Thermo Scientific), as previously described [29]. The LMP1 peptide, MSDWTGGAL-**C**-LWNLHGQAL (BioMer Technology, Pleasanton, CA, USA), comprises two of the three external loops of LMP1 [25], and is joined by a cysteine (bold) that provides linkage via the heterobifunctional linker SMCC, which has the potential to join one peptide to each of the 2130 coat proteins on the virus surface. The LMP-1 peptide was dissolved in dimethylformamide (DMF) at 10 mg/mL, and then mixed in a 5-fold molar excess with TMV:SMCC overnight at room temperature on a shaking incubator. TMV-LMP1 was then purified with PEG precipitation and analyzed with SDS-PAGE (4–20%, Tris-MOPS-SDS; Genscript, Piscataway, NJ, USA) followed by Coomassie blue staining to confirm the peptide conjugation efficiency.

Successful peptide conjugation was measured with the size shift of the coat protein using SDS-PAGE, and quantitation of the peptide loading was calculated with the band density ratio (BioRad, ImageQuant) of the top band (TMV + LMP1) compared to the band density of the bottom band (TMV only) at 84.8%. Quantitation of a peptide vaccine dose was calculated with the ratio of the mass of the peptide (2073 Da) to the mass of the TMV coat (17,624 Da) times the percent peptide loading (85%). The final peptide dose (in micrograms of peptide per micrograms of coat protein) was calculated to be 9.99% with the total mass. Pc3.1-LMP1 plasmid DNA for vaccination was prepared using QIAprep extraction (Qiagen, Germantown, MD, USA) and quantitated with UV spectrophotometry (Nanodrop).

### 2.5. Vaccination and Immune Response Measurement

All studies in mice were conducted in accordance with the Guide for the Care and Use of Laboratory Animals, and as described in IACUC-approved protocols at Touro University California. Age-matched female C3H/Hen mice (Charles River, Harlan, CA, USA) were randomly divided into vaccine groups. Prior to vaccination, 20 µg per mouse of the TMV-peptide conjugate (delivering 2 µg of peptide) was diluted into 50 µL of phosphate-buffered saline (PBS), then combined with 50 µL of an AddaVax adjuvant (InvivoGen) for subcutaneous injection. AddaVax is a squalene-based oil-in-water adjuvant known to support both antibody production and cellular activation [33]. In total, 50 µg of the pc3.1 LMP1 plasmid was used for intramuscular vaccination for each mouse, and was diluted with 50% AddaVax (InvivoGen) adjuvant and administered in 25 µL to each quadriceps muscle.

In the first experiment, mice received PBS control immunization, or a 20 µg dose of the TMV-LMP1 peptide fusion vaccine with subcutaneous administration, on days 0, 14, 28, and 42. Subcutaneous administration was consistent with previous 38C13 lymphoma vaccine testing in C3H mice [34]. On days 38 and 56, serum was collected from mice and tested for immune reactivity to the LMP1 peptide using ELISA. In the second experiment, mice were similarly injected with the PBS or TMV-LMP1 peptide vaccine with subcutaneous administration, or with 50 µg of pc3.1-LMP1 DNA with intramuscular administration. Three doses were given on days 0, 14, and 28, and serum was collected on day 42. For ELISA determination of antigen-specific immune responses, MAXISORP 96-well plates (ThermoFisher Scientific) were coated with an unconjugated LMP1 peptide antigen at a concentration of 5 µg/mL in 50 mM of a carbonate buffer (pH, 9.6). Additionally, 50 µL of 1 ng/µL of an unlabeled goat anti-mouse IgG antibody standard (Southern Biotech, 1030-01) was diluted three-fold in eight serial dilutions in the carbonate buffer in a single column of wells on each plate to serve as a quantitative IgG control. All coated plates were incubated at 4 °C overnight. An unbound capture peptide was removed from plates by washing three times with 200 µL of a solution of 1.5 M of NaCl and 0.5% Triton X-100. Plates were then incubated in a blocking solution (0.1 M of Tris (pH, 7.5), 2% BSA, 0.5% Tween 20) for 1 hour at room temperature. Mouse sera were diluted 1:10 in PBS with 2% BSA, diluted three-fold in eight serial dilutions, and incubated at room temperature for 1 hour. After incubation, plates were washed three times, as above, and a 1:5000 dilution of an HRP-conjugated goat anti-mouse IgG (H + L) secondary antibody (Southern Biotech, 1071-05) was added to each plate and incubated at room temperature for 1 hour. Plates were washed and then developed with TMB (Surmodics) and stopped with 0.5 M of H_2_SO_4_. Plates were read at 405 nm using a SpectraMax M5 plate reader and analyzed using SoftMax Pro software v6.3 (Molecular Devices, San Jose, CA, USA). Quantitation of LMP1-specific antibodies in serum was determined using the IgG internal standard on every plate.

The number of interferon-gamma (IFNγ)-secreting cells, a relevant measure of the potential for T cell activation, was tested with IFNγ ELISpot using single cell suspension of spleens from immunized mice, harvested 8 days after vaccine #4 in experiment 1 or after vaccine #3 in experiment 2. Membrane plates (MultiScreen IP; Millipore Sigma) were coated with anti-IFNγ antibodies as per the manufacturer’s protocol (BD Biosciences). Mouse splenocytes from four immunized mice were isolated under aseptic conditions, counted for viable cells (Vicell XR, Beckman-Coulter, Pasadena, CA, USA), diluted, and combined into a single cell suspension of 2 × 10^6^ spleen cells/mL from two mice. Antigen specific responses were measured by stimulating 2 × 10^5^ cells with peptide antigens (20 µg/mL), either the LMP1 full-length peptide MSDWTGGAL-**C**-LWNLHGQAL, MSDWTGGAL (LMP1 loop 1), or LWNLHGQAL (LMP1 loop 2). Phorbol myristate acetate/ionomycin (2.5 ng/mL, 250 ng/mL) was used as an assay positive control. Negative control cells were isolated from mice injected with PBS, or with cells only and no antigen stimulation. Irrelevant stimulation was with a B-gal specific peptide (ICPMYARV; [30]), which was also relevant to control for any potential non-specific effects of the cysteine in the full-length LMP1 peptide. Cells were stimulated for 36 h at 37 °C, 5% CO_2_, and 95% humidity, and then developed as per the manufacturer’s instructions. The reaction was stopped with distilled water, and plates were left to dry overnight. Spots were counted with an AID ELISpot plate reader (minimum diameter, 20 µm).

### 2.6. Tumor Challenge

38C13-LMP1 tumor cells (~50 × 10^6^ cells) were grown to a 2 × 10^6^ cell density in RPMI media (log phase growth) and then frozen in liquid nitrogen to generate a master cell bank for all challenge studies, to minimize batch specific influences on tumor outgrowth. In the tumor challenge experiments, 500 tumor cells (prepared with serial dilution) were given using subcutaneous administration 14 days after the last immunization, which localized the tumor under the skin, and provided an easily measured endpoint of the tumor size. Digital calipers were used to measure the length and width of the tumor mass at the longest and widest point of the tumor, starting at day 6, and thereafter for every 2 days until 5 days after no mice had a tumor, or at day 40. Specific humane endpoints were established at a maximum tumor area (length × width) of 2 cm, where tumors showed no evidence of necrosis, and mice exhibited normal feeding and grooming behaviors. Euthanasia was according to approved AVMA guidelines, using CO_2_ asphyxiation followed by cervical dislocation. 38C13-LMP1 transformed cells were tested for equivalent subcutaneous tumor growth to the parent 38C13 cell line. Both cell lines generated 50% survival at day 18 and 0% survival by day 30 using a Kaplan–Meier analysis, with a 200-cell initial subcutaneous dose, with statistically similar survival curves (*p* = 0.97).

### 2.7. Statistical Analysis

A statistical analysis was performed using Prism 8 software (GraphPad, San Diego, CA, USA) using Mann–Whitney tests for an ELISA analysis and a Kaplan–Meier log-rank survival analysis for a mouse tumor challenge analysis. Differences between groups or outcomes were considered statistically significant at *p* < 0.05.

## 3. Results

### 3.1. Validation of pc3.1-LMP1 Expression

**Figure 1:**
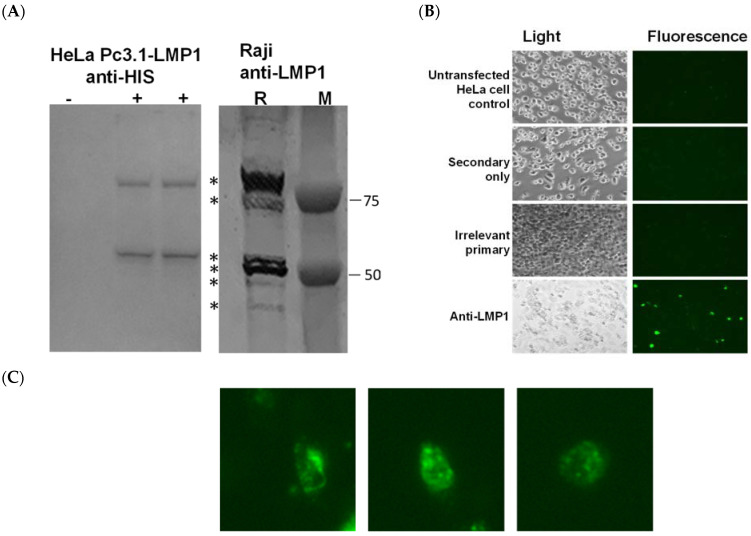
LMP1 expression confirmed with a Western analysis, or with cell surface staining of LMP1 transfected HeLa cells. (**A**) Extracts of nonntransfected HeLa cells (−) or HeLa cells transfected with pc3.1-LMP1 plasmid DNA (+) were prepared using lysis in a RIPA buffer after 24 h of incubation. SDS-PAGE separated proteins and a molecular weight marker (M; dual color, BioRad) were then transferred to the PVDF membrane and probed with rabbit-anti-HIS. Expression of LMP1 (*) is also shown in Raji cell extrac©(R) prepared using lysis in a RIPA buffer and probed with an anti-LMP1 primary antibody. (**B**) HeLa cells were seeded into 12-well plates and transfected with Pc3.1 LMP1 plasmid DNA. Immunocytochemistry was performed after 24 h using a secondary-only (Anti-mouse Alexa Fluor 488), irrelevant primary and secondary, or mouse monoclonal anti-LMP1 primary antibody with a secondary. (**C**) Expanded magnification (300×) of select cells, showing both cytoplasmic and membrane localization.

In order to create a B cell line expressing LMP1, we first validated CMV-driven expression of LMP1 protein expression with Pc3.1-LMP1 transfection into HeLa cells. LMP1 was detected with a Western blot at 54 Kd, the expected mass of the protein, as well as an apparent dimer, consistent with native LMP1 expression in human EBV-infected Raji cells (Figure 1A). However, far fewer bands were evident in the HeLa transfected sample than in native LMP1-expressing Raji cells. A further analysis in transfected cells confirmed expression in HeLa cells with anti-LMP1 immunofluorescence (Figure 1B). Although LMP1 is known to be a surface-exposed antigen, cell staining was observed in surface-exposed and intracellular localization, based on higher magnification images of fluorescence imaging (Figure 1C). Both western and immunofluorescence staining indicate that LMP1 full-length protein is expressed from the pc3.1 LMP1 DNA.

### 3.2. Transformation of 38C13 Mouse B Cells

**Figure 2:**
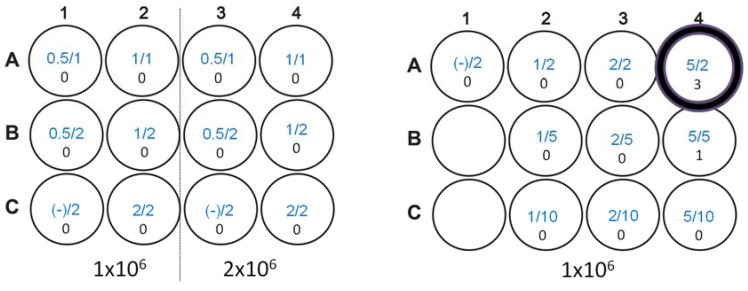
Tests of 38C13 Lipofectamine 2000 transfection efficiency using a GFP reporter plasmid.

Non-adherent B cell lines are notoriously difficult to transfect with standard methods [35]. However, in order to develop a mouse B cell model of EBV disease, successful transformation of the 38C13 B cell line was of paramount importance. We initially tested two different ratios of eGFP plasmid DNA to Lipofectamine 2000 (Figure 2, left panel, which shows the 12-well plate layout). Using DNA (first number in micrograms) and Lipofectamine 2000 (second number in microliters) in recommended ratios (0.5, 1, and 2 µg to µL) generated no GFP-positive cells (number below ratio). We evaluated alternative conditions outside the recommended range (1, 2, and 5 µg of DNA; 2, 5, and 10 µL of Lipofectamine) and determined that 1 × 10^6^ 38C13 cells transfected in 1 mL of Opti-MEM™ media with 2 µg of DNA using 5 µL of Lipofectamine could be used to select for pc3.1-LMP1 transformants based on improved GFP expression levels (Figure 2, right panel), from zero to three positive clones (circled). A 38C13 kill curve established that 500 µg/mL of G418 generated 100% cell death in 5 days, and was used for cell selection after transfection. After LMP1 DNA transformation, individual clones were observed at a very low frequency, at approximately four total clones out of a 6 × 10^6^ starting cell density, similar to the low number of positive cells after GFP plasmid transfection optimization.

**Figure 3:**
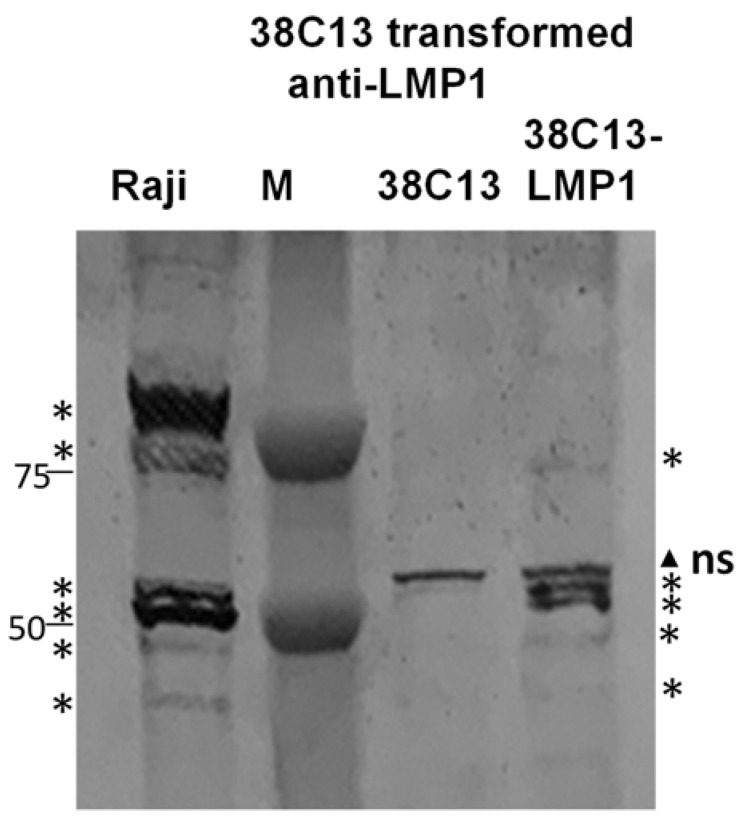
LMP1 is expressed in transformed 38C13 cells. 38C13 LMP1 transformed and native 38C13 cells were evaluated for LMP1 protein expression in comparison to native LMP1 expression in Raji cell extracts (human EBV-expressing), using an anti-LMP1 antibody. After 10 passages, extracts from the G9 transformed cell line were consistent with LMP1 protein expression compared to Raji cells. Specific LMP1 bands are marked with asterisks, and a non-specific reactive (ns) band in 38C13 cells is marked with a filled triangle.

Protein expression of LMP1 was verified in 38C13 clones with a Western blot (Figure 3). Although transfection of this plasmid into HeLa cells generated only a monomer and dimer of LMP1 (Figure 1), 38C13 transformed cells more closely resembled Raji cell extracts expressing native LMP1, with multiple bands at 50 Kd and 100 Kd (Figure 3 asterisks). 38C13-LMP1 cells also had multiple bands at 50 Kd, but had less evident higher molecular weight species. Of note, the anti-LMP1 antibody (Sigma MABF2248) also recognized non-specific bands (Figure 3 triangle) that were present in non-transformed 38C13 cells, which was distinct from the specific bands that are consistent with Raji cell native LMP1 (Figure 3 asterisks). Similar specific and non-specific bands were visible in the cytosolic extract (Figure S2). After approximately 10 passages, three clones expressed LMP1 with an anti-LMP1 Western blot. One clone, G9, had stable expression of the LMP1 protein and was designated as 38C13-LMP1, as shown in Figure 3.

### 3.3. Creation of an LMP1 Peptide Vaccine

**Figure 4:**
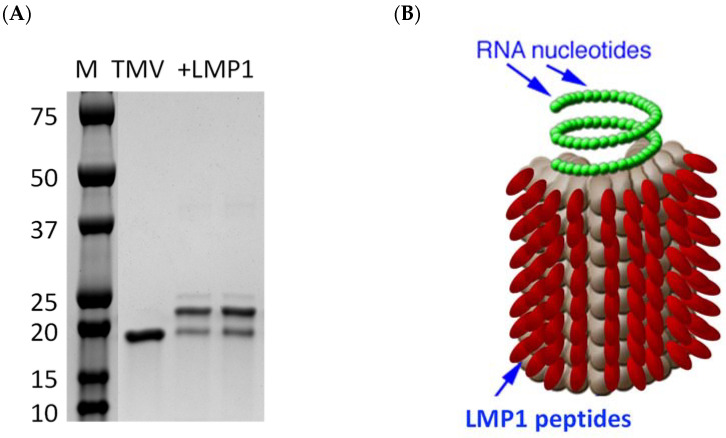
TMV-LMP1 fusion peptide vaccine. (**A**) The TMV virus (TMV) expressing a surface exposed lysine was activated by SMCC, and then incubated with an LMP1 peptide overnight at room temperature. Samples were then analyzed with SDS-PAGE for the size shift, with the ratio of the conjugated TMV monomer (arrow) and dimer (arrowhead) to the unconjugated TMV coat (filled circle) being 85%. (**B**) TMV-LMP1 peptide fusion vaccine schematic, representing the viral display of peptides.

In order to develop the second component of the EBV-specific mouse vaccine immunotherapy model, we created a peptide fusion of two extracellular loops of LMP1 [25] onto the surface of a plant-produced Tobacco Mosaic virus (TMV), which is an effective carrier for peptides in cancer vaccination settings. TMV provides a nanoparticle size and repeated antigen display for increasing antigen uptake with APCs, and viral plus strand RNA enhances antibody and cell-mediated immune activation [29,30,31]. TMV-LMP1 fusion increases the mass of TMV coat protein (17,484) with a shift in the protein mass equivalent to the peptide mass (2072) using SDS-PAGE (Figure 4A). Although the SDS-PAGE analysis indicates a single-coat protein fused to an individual peptide, the TMV virus is a rod-shaped virion of 2130 assembled coat proteins around an RNA core. With about 85% coat proteins loaded with a peptide, each virus will carry a high density of approximately 1800 peptide antigens per virus particle, represented by a schematic of 100% peptide loading onto TMV (Figure 4B).

### 3.4. Immunization, Immune Analysis, and Tumor Challenge

**Figure 5:**
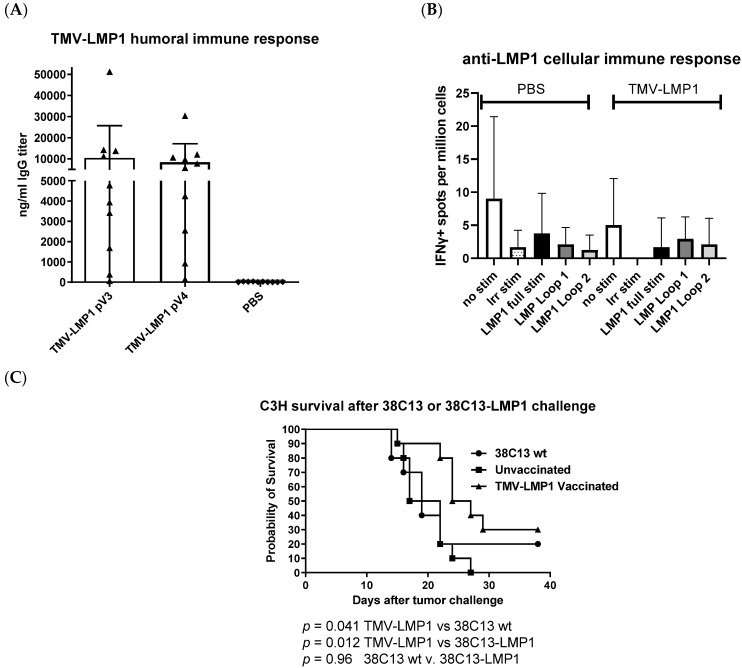
Immunity and tumor protection after TMV-LMP1 peptide vaccination. (**A**) Serum was collected from TMV-LMP1 immunized mice and tested for specific anti-LMP1 IgG titers with ELISA. (**B**) Single cell suspensions of spleen cells from 2 mice were independently pooled and tested with IFNγ ELISpot. In total, 2 × 10^5^ cells were stimulated with 20 µg/mL of a peptide, either full-length (MSDWTGGAL-**C**-LWNLHGQAL), MSDWTGGAL (loop 1), or LWNLHGQAL (loop 2). The B-gal peptide ICPMYARV was used as a non-specific stimulation (Irr stim). (**C**) Vaccinated (triangle) or naive (square) mice were challenged subcutaneously with 500 38C13-LMP1 cells or with 500 38C13 wild-type (wt, circle) cells, and followed for 40 days for tumor growth. Study endpoints were set at 2 cm of the tumor area or tumor necrosis for all mice. Survival was plotted using a Kaplan–Meier log-rank analysis (Prism, Graphpad).

To establish if LMP1 vaccination would stimulate appropriate anti-LMP1 immunity, we vaccinated 10 C3H/He (syngeneic with the 38C13 cell line) with about 2 µg of a peptide in 20 µg of the vaccine using subcutaneous administration every 2 weeks for a total of four doses. Ten days after immunizations #3 and #4, we measured antibody responses to the LMP1 peptide, as shown in Figure 5A. After three doses, all but one animal had a measurable anti-LMP1 immune response. Although a fourth dose was administered, no additional boosting was achieved, and the median titer of about 10,000 ng/mL was not statistically different between dose 3 and dose 4. In a parallel experiment, five mice per group were immunized (Figure 5A) with either the PBS control or TMV-LMP1 vaccine. Although the vaccine peptide sequences did not contain predicted H2k CTL epitopes [36,37], H2k epitopes are not as well characterized as for other MHC types in mice or human HLA. So, to directly determine the potential for cytotoxic T lymphocyte (CTL) activation, we tested for cellular responses that might occur through cryptic MHC binding [38], or other cellular response pathways. We tested secretion of interferon gamma (IFNγ) as a general marker for cellular activation, which includes T cells, natural killer (NK) cells, and macrophages [39]. Eight days after vaccine 4, we tested for IFNγ secretion with ELISpot using spleen single cell suspensions from vaccinated mice. Cells were stimulated with the LMP1 vaccine peptide, loop 1 peptide, or loop 2 peptide for 36 h and then positive spots were counted and normalized to 1 × 10^6^ cells. Although control (PMA/ionomycin)-stimulated cells were evident (too numerous to count) for both PBS- and TMV-LMP1-vaccinated mice, no cellular responses were measured after LMP1 peptide stimulation (Figure 5B).

Mice vaccinated for antibody testing were then challenged 14 days after the last immunization with subcutaneous administration of 38C13-LMP1 tumor cells. A subcutaneous depot of cells form a tumor mass that can be measured with calipers. Mice were followed for at least 35 days, or 5 days after the last tumor death (a defined endpoint of 2 × 1 cm in area). As shown in Figure 5C, mice immunized with the TMV-LMP1 peptide vaccine were significantly protected from the 38C13-LMP1 tumor challenge (*p* = 0.012), compared to the PBS-immunized control group, or compared to the survival of mice challenged with wild-type 38C13 cells (*p* = 0.042). There were no differences in the cell growth rate between wild-type and LMP1-transformed 38C13 tumors (*p* = 0.96).

**Figure 6:**
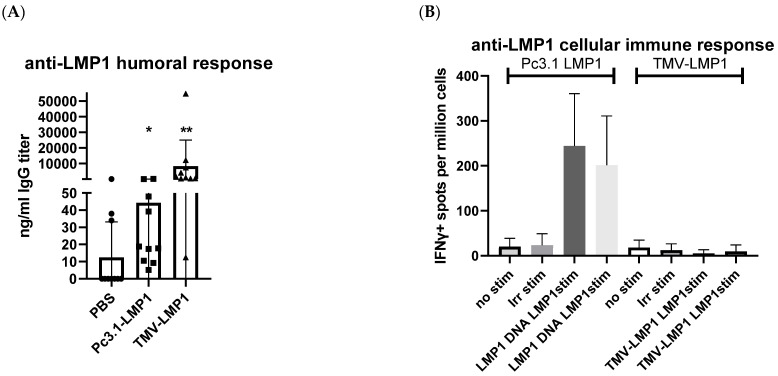

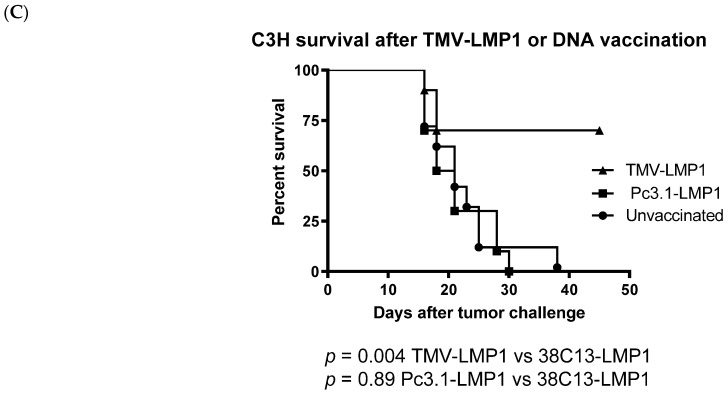
The TMV-peptide vaccine and plasmid DNA vaccine induce quantitatively different antibody responses, and tumor protection. (**A**) Both the TMV-LMP1 vaccine and Pc3.1-LMP1 plasmid DNA vaccine (squares) stimulate significant IgG antibody responses compared to PBS (circles) control mice (** *p* < 0.001 and * *p* = 0.02, respectively). (**B**) Single cell suspensions of spleen cells from four mice, two pools of two mice each, were tested with IFNγ ELISpot. In total, 2 × 10^5^ cells were stimulated with 20 µg/mL of a peptide (MSDWTGGAL-**C**-LWNLHGQAL) and then analyzed for IFNγ secretion. (**C**) TMV-LMP1 peptide vaccine mice, LMP1 DNA vaccine mice, and PBS control mice were inoculated subcutaneously with 500 38C31-LMP1 cells, and solid tumors were measured using calipers. At day 35, the survival of the TMV-LMP1 vaccine group demonstrates significantly better survival (*p* = 0.021) than the parent cell line, while the LMP1 plasmid DNA vaccine group does not have any protective effect (*p* = 0.89). Study endpoints were set at a 200 mm^2^ tumor volume or tumor necrosis for all mice. Survival was plotted using a Kaplan–Meier log-rank analysis (Prism, Graphpad).

Because IgG titers were not boosted by a fourth immunization, we then tested survival after three vaccine doses. We also tested if immunization with pc3.1-LMP1 plasmid DNA, potentially encoding more diverse epitopes, could stimulate broader humoral and cellular immunity that may enhance tumor protection. DNA vaccines are known to be able to stimulate stronger cellular immune responses due to the DNA uptake by the host APCs, allowing the whole antigen to be expressed and the immunostimulatory epitopes to be presented to CD4+ and CD8+ T cells [40]. Since this was the same DNA that was used for transfections into HeLa cells, and the same DNA used to make the 38C13-LMP1 cell line, the DNA plasmid vaccine has both a confirmed expression and relevance to the transformed tumor cell line. In the second study, we repeated immunization of 10 mice per group for the challenge, and in a separate study, 5 mice per group for a cellular analysis. Figure 6A shows that serum mean antibody titers after three doses of TMV-LMP1 were equivalent to the previous experiment, at around 10,000 ng/mL, and were statistically greater than PBS (*p* < 0.001) and DNA vaccination (*p* = 0.0062). Consistent with many prior studies of DNA immunization in mice, IgG titers were significantly lower after Pc3.1-LMP1 DNA immunization, though still significantly higher than PBS (*p* = 0.02). In contrast, DNA immunization improved anti-LMP1 IFNγ ELISpot levels after LMP1 peptide stimulation to low but measurable titers, where TMV-LMP1 peptide immunization again only resulted in background titers (Figure 6B) after stimulation with the full-length LMP1 peptide antigen for 36 h. Because of these results, a more detailed T cell analysis was not initiated. Mice immunized for an antibody titer analysis (Figure 6A) were challenged 8 days after the last vaccine dose, and followed for tumor growth. As shown in Figure 6C, mice immunized with TMV-LMP1 were significantly protected from tumor growth compared to the PBS-negative control-vaccinated mice (*p* = 0.021), or the LMP1 DNA vaccine. Despite the improved cellular responses, the LMP1 DNA vaccine was unable to protect mice against the 38C13-LMP1 challenge, and survival curves were not different than the PBS control immunization group (*p* = 0.89).

## 4. Discussion

EBV is a ubiquitous virus with a prevalence of up to 90% among the worldwide population, and nearly 200,000 new cases of EBV-associated diseases are diagnosed each year [24]. EBV is implicated in a variety of malignant lymphoproliferative disorders including Hodgkin’s lymphoma, DLBCL, NPC, GC, and post-transplant and HIV-related lymphomas, and therefore represents an ongoing public health concern. To study EBV-associated lymphoma in a mouse model, researchers have used severe combined immunodeficient (SCID) mice and humanized mice implanted with human hematopoietic stem cells to replicate a human immune response as closely as possible [41]. However, these types of xenograft mouse models are not suited for testing vaccine immunotherapy due to limitations in fully simulating a complete immune response [23]. To overcome these limitations, we created a mouse B cell line that was transformed with the EBV latency antigen LMP1, which is an antigen that is expressed in nearly all stages of EBV disease [7,42]. This cell line can be used to test vaccine efficacy and establish mechanisms of protection against a tumor challenge. Although transfection and selection have been shown to be an effective method of generating stable mouse cell lines, no one has yet established a true B cell mouse model capable of being used to investigate potential vaccine treatments against EBV disease specifically associated with lymphoma. Other groups have transformed TC-1 cells with EBV antigens [43,44], but this cell line is derived from primary papillomavirus-transformed lung epithelial cells of C57BL/6 mice, which may not adequately represent antigen presentation in a context most relevant to B cell disease, particularly since B cells are also antigen-presenting cells with a unique antigen processing capability.

The objectives of our study were to use DNA transfection and transformation to develop a mouse B cell tumor line expressing LMP1, and to generate protective immunity in mice vaccinated with a plasmid DNA or a peptide LMP1 antigen fused to TMV. Based on both a Western blot (Figure 1) and LMP1 cell immunocytochemistry (Figure 3), we concluded that plasmid transfection was able to induce antigen expression. Transfection with the same LMP1 plasmid and G418 selection was then used to establish 38C13 clones expressing LMP1 protein. Interestingly, a Western blot analysis revealed that 38C13 cells expressed multiple LMP1 species more similar to LMP1 expressed by Raji cells, in contrast to the same construct expressed in HeLa cells, which was most likely the result of B cell-specific post-translational modification, or site-specific proteolysis. Also of note, although LMP1 is thought to be a surface-expressed antigen, we were able to visualize surface expression of the protein in transfected HeLa cells (Figure 3), and we also observed LMP1 cytoplasmic expression. However, cytoplasmic expression was similar to that seen in LMP1-expressing human Hodgkin’s tumors [45] and in human NPC [13].

TMV-peptide fusion vaccines have been used successfully in cancer vaccine applications, and multi-antigen fusions were more successful than other forms of immunization against aggressive murine melanoma [29,31], supporting the concept of a multivalent vaccine formulation. TMV has proven APC uptake and immune activation after in vivo administration [30], and was considered as an attractive antigen delivery system for the development of an EBV vaccine. Immunization with the TMV-LMP1 peptide epitope vaccine (Figure 4) was able to stimulate a significant IgG antibody response, but was not able to induce a cellular response as measured by IFNγ ELISpot (Figure 5). After vaccination, C3H mice were challenged with 38C13-LMP1, and the TMV-LMP1 peptide epitope vaccine conferred protective immunity after four doses of the vaccine (Figure 5). Since IgG titers did not improve after three vaccine doses, we repeated the study with three TMV-LMP1 peptide vaccines, and compared immune protection to full-length plasmid pc3.1 LMP1 DNA vaccination, which may provide more antigens and better cellular activation. Given that the LMP1 loop epitopes were the primary stimulation for IFNγ secretion, there may be additional T cell antigens that could be present after DNA immunization that were not detected by this analysis. We were able to definitively show that the pc3.1 LMP1 plasmid DNA vaccine did induce a cellular response, using IFNγ ELISpot, but this was not sufficient to protect mice, where the TMV-LMP1 peptide vaccine had confirmed protection in two separate studies (Figure 6). This is in contrast to protection in two different studies using an epithelial TC-1 LMP1-expressing cell line [28,44]. This difference may be due to the MHC restriction of antigens that differs between C3H (H2k) and TC-1 syngeneic C57/BL6 (H2b) mouse strains, or the potential for an enhanced efficacy of NK cell killing in cells like 38C13 that are MHC II-deficient [46]. This is especially relevant to human disease, since EBV plus B cell tumors that lack MHC II antigen presentation predict a poor outcome after standard treatment [47]. In this study, antibody responses to LMP1 seem to be the only protective mechanism for survival, which is consistent with use of this B cell line to investigate the protective efficacy of 38C13 idiotype immunization [34,48,49]. This is also consistent with current highly successful antibody-based therapies against human B cell lymphoma, such as Rituximab, which targets CD20 on the cell surface. Future studies can model the mechanism of protection by the adoptive transfer of antibodies, and the potential roles of antibody-dependent cellular cytotoxicity (ADCC) and natural killer (NK) cells in tumor control. In addition, we will combine therapeutic vaccination using LMP1 antigens with 38C13 idiotype vaccination, which can also partially protect mice against a 38C13 tumor challenge [34,48,49]. More importantly, idiotype vaccination may present another pathway to improve immunotherapy in human patients [50,51], in combination with anti-EBV vaccination. In addition, we can continue to explore the use of 38C13 cell transformation for the study of other EBV antigen vaccines like LMP2, which is also a surface-expressed antigen, and the nuclear antigen EBNA1, in order to validate new protective antigens and establish combination vaccines targeting EBV+ tumor cells. TMV has also been shown to be an excellent carrier for whole antigen vaccination against other viral disease antigens [52,53,54], so testing whole antigen fusions for LMP1, LMP2, and EBNA may be considered in the future. Lastly, it is now evident that checkpoint inhibitors are amplified in some forms of EBV disease [55], and combined anti-checkpoint immunotherapy may also improve clinical outcomes.

Our future goals will be to generate a multi-antigen-expressing B cell line that will facilitate the study of antigen-specific vaccination against EBV that covers the latency periods I, II, and III [56], which would offer broader vaccine immunotherapeutic strategies, including full-length antigens, with combined cellular and antibody targets specific for EBV-associated diseases. Additional studies will also include the evaluation of natural killer (NK) cell activation, which may work in concert with antigen-specific antibodies to eradicate tumors [57,58]. A second priority will be to test multivalent vaccine efficacy in a therapeutic mouse model of disease that more closely reflects the difficulty of treating EBV-related disease in patients. A multivalent EBV vaccine would be consistent with current approaches to EBV-associated lymphomas, which take advantage of a wide range of treatment modalities, including traditional chemotherapy, antibody therapy, adoptive cell transfer (ACT), chimeric antigen receptor T cell (CAR-T) therapy, and vaccination [24,41]. While effective, ACT and CAR-T therapies are time-consuming to produce and can be prohibitively expensive for patients. Expanding the current knowledge of potential vaccine immunotherapies for malignancies will benefit our understanding of alternate methods of targeting EBV-associated lymphomas.

## 5. Conclusions

Our study accomplished two important goals, the development of a murine B cell model of EBV disease and the demonstration of improved immune protection against a tumor challenge using a novel LMP-1 peptide antigen delivery system based on the plant virus TMV. Interestingly, we show that antibody responses to the surface-expressed LMP-1 peptide are sufficient for protection. We propose, to further develop this model system, testing vaccination in a multi-antigen-expressing B cell tumor, where both the antibody and cellular response can protect mice against a tumor challenge, especially in a therapeutic vaccine model.

## Data Availability

All of the data collected in this study are presented, no additional data is available as supporting material.

## Supplementary Materials

The following supporting information can be downloaded at: https://www.mdpi.com/article/10.3390/biology12070983/s1, Figure S1: Original Western blot of HeLa cell LMP1 transfected RIPA extracts; Figure S2: Original Western blot of LMP1 transformed 38C13 cells.

## References

[1] Munz C. (2019). Latency and lytic replication in Epstein-Barr virus-associated oncogenesis. Nat. Rev. Microbiol..

[2] Maeda E., Akahane M., Kiryu S., Kato N., Yoshikawa T., Hayashi N., Aoki S., Minami M., Uozaki H., Fukayama M. (2009). Spectrum of Epstein-Barr virus-related diseases: A pictorial review. Jpn. J. Radiol..

[3] Cui X., Snapper C.M. (2021). Epstein Barr Virus: Development of Vaccines and Immune Cell Therapy for EBV-Associated Diseases. Front. Immunol..

[4] Zheng X., Huang Y., Li K., Luo R., Cai M., Yun J. (2022). Immunosuppressive Tumor Microenvironment and Immunotherapy of Epstein-Barr Virus-Associated Malignancies. Viruses.

[5] Wong Y., Meehan M.T., Burrows S.R., Doolan D.L., Miles J.J. (2022). Estimating the global burden of Epstein-Barr virus-related cancers. J. Cancer Res. Clin. Oncol..

[6] Soldan S.S., Lieberman P.M. (2023). Epstein-Barr virus and multiple sclerosis. Nat. Rev. Microbiol..

[7] Saha A., Robertson E.S. (2011). Epstein-Barr virus-associated B-cell lymphomas: Pathogenesis and clinical outcomes. Clin. Cancer Res..

[8] Shannon-Lowe C., Rickinson A.B., Bell A.I. (2017). Epstein–Barr virus-associated lymphomas. Philos. Trans. R. Soc. B Biol. Sci..

[9] Williams H. (2005). Epstein-Barr virus: The impact of scientific advances on clinical practice. Blood.

[10] Chen J., Hu C.-F., Hou J.-H., Shao Q., Yan L.-X., Zhu X.-F., Zeng Y.-X., Shao J.-Y. (2010). Epstein-Barr virus encoded latent membrane protein 1 regulates mTOR signaling pathway genes which predict poor prognosis of nasopharyngeal carcinoma. J. Transl. Med..

[11] Kwon J., Park Y., Kang J., Kim K., Ko Y., Ryoo B., Lee S., Lee S., Koo H., Kim W. (2006). The effect of Epstein–Barr virus status on clinical outcome in Hodgkin’s lymphoma. Ann. Hematol..

[12] Hariwiyanto B., Sastrowiyoto S., Mubarika S., Salugu M. (2010). LMP1 and LMP2 may be prognostic factors for outcome of therapy in nasopharyngeal cancers in Indonesia. Asian Pac. J. Cancer Prev..

[13] Zhao Y., Wang Y., Zeng S., Hu X. (2012). LMP1 expression is positively associated with metastasis of nasopharyngeal carcinoma: Evidence from a meta-analysis. J. Clin. Pathol..

[14] Graham J.P., Arcipowski K.M., Bishop G.A. (2010). Differential B-lymphocyte regulation by CD40 and its viral mimic, latent membrane protein 1. Immunol. Rev..

[15] Higuchi M., Izumi K.M., Kieff E. (2001). Epstein–Barr virus latent-infection membrane proteins are palmitoylated and raft-associated: Protein 1 binds to the cytoskeleton through TNF receptor cytoplasmic factors. Proc. Natl. Acad. Sci. USA.

[16] D’Souza B.N., Edelstein L.C., Pegman P.M., Smith S.M., Loughran S.T., Clarke A., Mehl A., Rowe M., Gelinas C., Walls D. (2004). Nuclear Factor B-Dependent Activation of the Antiapoptotic bfl-1 Gene by the Epstein-Barr Virus Latent Membrane Protein 1 and Activated CD40 Receptor. J. Virol..

[17] Liebowitz D., Mannick J., Takada K., Kieff E. (1992). Phenotypes of Epstein-Barr virus LMP1 deletion mutants indicate transmembrane and amino-terminal cytoplasmic domains necessary for effects in B-lymphoma cells. J. Virol..

[18] Mancao C., Hammerschmidt W. (2007). Epstein-Barr virus latent membrane protein 2A is a B-cell receptor mimic and essential for B-cell survival. Blood.

[19] Vrzalikova K., Ibrahim M., Nagy E., Vockerodt M., Perry T., Wei W., Woodman C., Murray P. (2018). Co-Expression of the Epstein-Barr Virus-Encoded Latent Membrane Proteins and the Pathogenesis of Classic Hodgkin Lymphoma. Cancers.

[20] Rowe M. (1991). Epstein-Barr virus (EBV)-associated lymphoproliferative disease in the SCID mouse model: Implications for the pathogenesis of EBV-positive lymphomas in man. J. Exp. Med..

[21] Yajima M., Imadome K.I., Nakagawa A., Watanabe S., Terashima K., Nakamura H., Ito M., Shimizu N., Honda M., Yamamoto N. (2008). A New Humanized Mouse Model of Epstein-Barr Virus Infection That Reproduces Persistent Infection, Lymphoproliferative Disorder, and Cell-Mediated and Humoral Immune Responses. J. Infect. Dis..

[22] Yajima M., Imadome K.I., Nakagawa A., Watanabe S., Terashima K., Nakamura H., Ito M., Shimizu N., Yamamoto N., Fujiwara S. (2009). T Cell–Mediated Control of Epstein-Barr Virus Infection in Humanized Mice. J. Infect. Dis..

[23] Walsh N.C., Kenney L.L., Jangalwe S., Aryee K.-E., Greiner D.L., Brehm M.A., Shultz L.D. (2017). Humanized Mouse Models of Clinical Disease. Annu. Rev. Pathol..

[24] Cohen J.I., Fauci A.S., Varmus H., Nabel G.J. (2011). Epstein-Barr Virus: An Important Vaccine Target for Cancer Prevention. Sci. Transl. Med..

[25] Delbende C., Verwaerde C., Mougel A., Tranchand Bunel D. (2009). Induction of Therapeutic Antibodies by Vaccination against External Loops of Tumor-Associated Viral Latent Membrane Protein. J. Virol..

[26] Longnecker R., Druker B., Roberts T.M., Kieff E. (1991). An Epstein-Barr virus protein associated with cell growth transformation interacts with a tyrosine kinase. J. Virol..

[27] Meij P., Leen A., Rickinson A.B., Verkoeijen S., Vervoort M.B.H.J., Bloemena E., Middeldorp J.M. (2002). Identification and prevalence of CD8+ T-cell responses directed against Epstein-Barr virus-encoded latent membrane protein 1 and latent membrane protein 2. Int. J. Cancer.

[28] Lin M.C., Lin Y.C., Chen S.T., Young T.H., Lou P.J. (2017). Therapeutic vaccine targeting Epstein-Barr virus latent protein, LMP1, suppresses LMP1-expressing tumor growth and metastasis in vivo. BMC Cancer.

[29] McCormick A.A., Corbo T.A., Wykoff-Clary S., Nguyen L.V., Smith M.L., Palmer K.E., Pogue G.P. (2006). TMV-peptide fusion vaccines induce cell-mediated immune responses and tumor protection in two murine models. Vaccine.

[30] Kemnade J.O., Seethammagari M., Collinson-Pautz M., Kaur H., Spencer D.M., McCormick A.A. (2014). Tobacco mosaic virus efficiently targets DC uptake, activation and antigen-specific T cell responses in vivo. Vaccine.

[31] McCormick A.A., Corbo T.A., Wykoff-Clary S., Palmer K.E., Pogue G.P. (2006). Chemical conjugate TMV-peptide bivalent fusion vaccines improve cellular immunity and tumor protection. Bioconjugate Chem..

[32] Smith M.L., Lindbo J.A., Dillard-Telm S., Brosio P.M., Lasnik A.B., McCormick A.A., Nguyen L.V., Palmer K.E. (2006). Modified tobacco mosaic virus particles as scaffolds for display of protein antigens for vaccine applications. Virology.

[33] Pattnaik A., Sahoo B.R., Struble L.R., Borgstahl G.E.O., Zhou Y., Franco R., Barletta R.G., Osorio F.A., Petro T.M., Pattnaik A.K. (2023). A Ferritin Nanoparticle-Based Zika Virus Vaccine Candidate Induces Robust Humoral and Cellular Immune Responses and Protects Mice from Lethal Virus Challenge. Vaccines.

[34] McCormick A.A., Kumagai M.H., Hanley K., Turpen T.H., Hakim I., Grill L.K., Tuse D., Levy S., Levy R. (1999). Rapid production of specific vaccines for lymphoma by expression of the tumor-derived single-chain Fv epitopes in tobacco plants. Proc. Natl. Acad. Sci. USA.

[35] Ling Yong C.L., Siak-Wei Ow D., Tandiono T., Mei Heng L.L., Kwok-Keung Chan K., Ohl C.D., Klaseboer E., Ohl S.W., Boon-Hwa Choo A. (2014). Microbubble-mediated sonoporation for highly efficient transfection of recalcitrant human B- cell lines. Biotechnol. J..

[36] Malik A., Houghten R., Corradin G., Buus S., Berzofsky J.A., Hoffman S.L. (1995). Identification of a nonameric H-2Kk-restricted CD8+ cytotoxic T lymphocyte epitope on the Plasmodium falciparum circumsporozoite protein. Infect. Immun..

[37] Millrain M., Scott D., Addey C., Dewchand H., Ellis P., Ehrmann I., Mitchell M., Burgoyne P., Simpson E., Dyson J. (2005). Identification of the immunodominant HY H2-D(k) epitope and evaluation of the role of direct and indirect antigen presentation in HY responses. J. Immunol..

[38] Vitiello A., Sette A., Yuan L., Farness P., Southwood S., Sidney J., Chesnut R.W., Grey H.M., Livingston B. (1997). Comparison of cytotoxic T lymphocyte responses induced by peptide or DNA immunization: Implications on immunogenicity and immunodominance. Eur. J. Immunol..

[39] Le Page C., Genin P., Baines M.G., Hiscott J. (2000). Interferon activation and innate immunity. Rev. Immunogenet..

[40] Lee S.-H., Danishmalik S.N., Sin J.-I. (2015). DNA vaccines, electroporation and their applications in cancer treatment. Hum. Vaccines Immunother..

[41] Pei Y., Lewis A.E., Robertson E.S. (2017). Current Progress in EBV-Associated B-Cell Lymphomas. Adv. Exp. Med. Biol..

[42] Kang M.S., Kieff E. (2015). Epstein-Barr virus latent genes. Exp. Mol. Med..

[43] Sun L., Hao Y., Wang Z., Zeng Y. (2018). Constructing TC-1-GLUC-LMP2 Model Tumor Cells to Evaluate the Anti-Tumor Effects of LMP2-Related Vaccines. Viruses.

[44] Wojtak K., Perales-Puchalt A., Weiner D.B. (2019). Novel Synthetic DNA Immunogens Targeting Latent Expressed Antigens of Epstein-Barr Virus Elicit Potent Cellular Responses and Inhibit Tumor Growth. Vaccines.

[45] Hashmi A.A., Hussain Z.F., Hashmi K.A., Zafar M.I., Edhi M.M., Faridi N., Khan M. (2017). Latent membrane protein 1 (LMP1) expression in Hodgkin lymphoma and its correlation with clinical and histologic parameters. World J. Surg. Oncol..

[46] Li Y., Wang X., Cheng C., Jiang S., Ma T., Xu L. (2020). Absence of MHC class Ⅱ molecules promotes natural killer cells activation in mice. Int. Immunopharmacol..

[47] Jiang X.N., Yu B.H., Yan W.H., Lee J., Zhou X.Y., Li X.Q. (2020). Epstein-Barr virus-positive diffuse large B-cell lymphoma features disrupted antigen capture/presentation and hijacked T-cell suppression. Oncoimmunology.

[48] Campbell M.J., Carroll W., Kon S., Thielemans K., Rothbard J.B., Levy S., Levy R. (1987). Idiotype vaccination against murine B cell lymphoma. Humoral and cellular responses elicited by tumor-derived immunoglobulin M and its molecular subunits. J. Immunol..

[49] Kwak L.W., Young H.A., Pennington R.W., Weeks S.D. (1996). Vaccination with syngeneic, lymphoma-derived immunoglobulin idiotype combined with granulocyte/macrophage colony-stimulating factor primes mice for a protective T-cell response. Proc. Natl. Acad. Sci. USA.

[50] Hsu F.J., Caspar C.B., Czerwinski D., Kwak L.W., Liles T.M., Syrengelas A., Taidi-Laskowski B., Levy R. (1997). Tumor-specific idiotype vaccines in the treatment of patients with B- cell lymphoma--long-term results of a clinical trial. Blood.

[51] McCormick A.A., Reddy S., Reinl S.J., Cameron T.I., Czerwinski D., Vojdani F., Hanley K.M., Garger S.J., White E.L., Novak J. (2008). Plant-produced Idiotype Vaccines for the Treatment of Non-Hodgkin’s Lymphoma: Safety and Immunogenicity in a Phase I Clinical Study. Proc. Natl. Acad. Sci. USA.

[52] Mallajosyula J.K., Jeevan T., Chickwamba R., Webby R.J., McCormick A.A. (2016). A single dose TMV-HA vaccine protects mice from H5N1 influenza challenge. Int. J. Vaccine Res..

[53] Mallajosyula J.K., Hiatt E., Hume S., Johnson A., Jeevan T., Chikwamba R., Pogue G.P., Bratcher B., Haydon H., Webby R.J. (2013). Single-dose monomeric HA subunit vaccine generates full protection from influenza challenge. Hum. Vaccines Immunother..

[54] Royal J.M., Simpson C.A., McCormick A.A., Phillips A., Hume S., Morton J., Shepherd J., Oh Y., Swope K., DeBeauchamp J.L. (2021). Development of a SARS-CoV-2 Vaccine Candidate Using Plant-Based Manufacturing and a Tobacco Mosaic Virus-like Nano-Particle. Vaccines.

[55] Green M.R., Rodig S., Juszczynski P., Ouyang J., Sinha P., O’Donnell E., Neuberg D., Shipp M.A. (2012). Constitutive AP-1 activity and EBV infection induce PD-L1 in Hodgkin lymphomas and posttransplant lymphoproliferative disorders: Implications for targeted therapy. Clin. Cancer Res..

[56] Ruhl J., Leung C.S., Munz C. (2020). Vaccination against the Epstein-Barr virus. Cell Mol. Life Sci..

[57] Gauthier L., Morel A., Anceriz N., Rossi B., Blanchard-Alvarez A., Grondin G., Trichard S., Cesari C., Sapet M., Bosco F. (2019). Multifunctional Natural Killer Cell Engagers Targeting NKp46 Trigger Protective Tumor Immunity. Cell.

[58] Wang W., Erbe A.K., Hank J.A., Morris Z.S., Sondel P.M. (2015). NK Cell-Mediated Antibody-Dependent Cellular Cytotoxicity in Cancer Immunotherapy. Front. Immunol..

