# Supplementary material for: Vaccination against Epstein–Barr Latent Membrane Protein 1 Protects against an Epstein–Barr Virus-Associated B Cell Model of Lymphoma
Source: Biology (Basel). 2023 Jul 11;12(7):983. doi: 10.3390/biology12070983 (PMC10376452; doi:10.3390/biology12070983)
Supplement: Supplementary file 1 [file biology-12-00983-s001.zip › biology-2347095-supplementary.pdf]

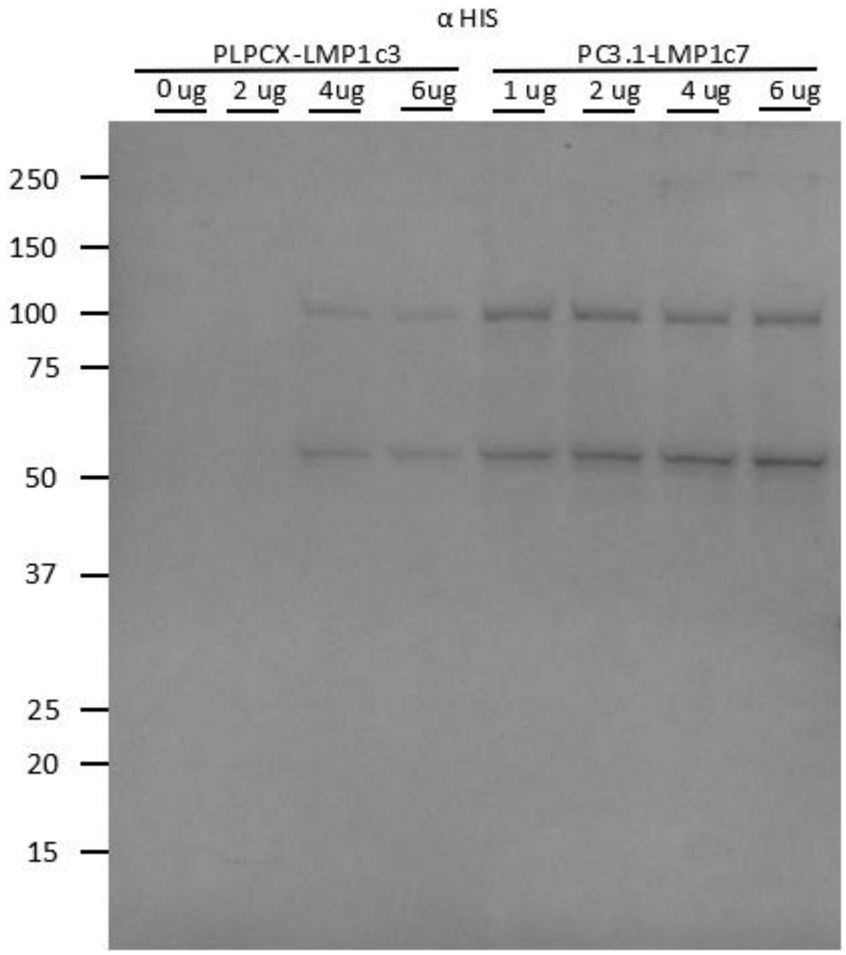


**Figure S1.** Original Western blot of HeLa cell LMP1 transfected RIPA extracts. $2 \times 10^6$ cells were prepared using 200 μL of a RIPA buffer 24 hours after transfection. 40 μL of protein extracts was separated with SDS-PAGE, transferred to PDVF membranes, and then probed rabbit anti-HIS (Genscript A00174-40) at 1:1000 dilution, followed by a goat anti-rabbit HRP secondary (Southern Biotech), and then developed with a TMB membrane substrate (Surmodics). Also shown are PLPCX-LMP1 viral transformed HeLa cells, which due to inferior transformation were not pursued further for creating LMP1 expressing 38C13 cells.

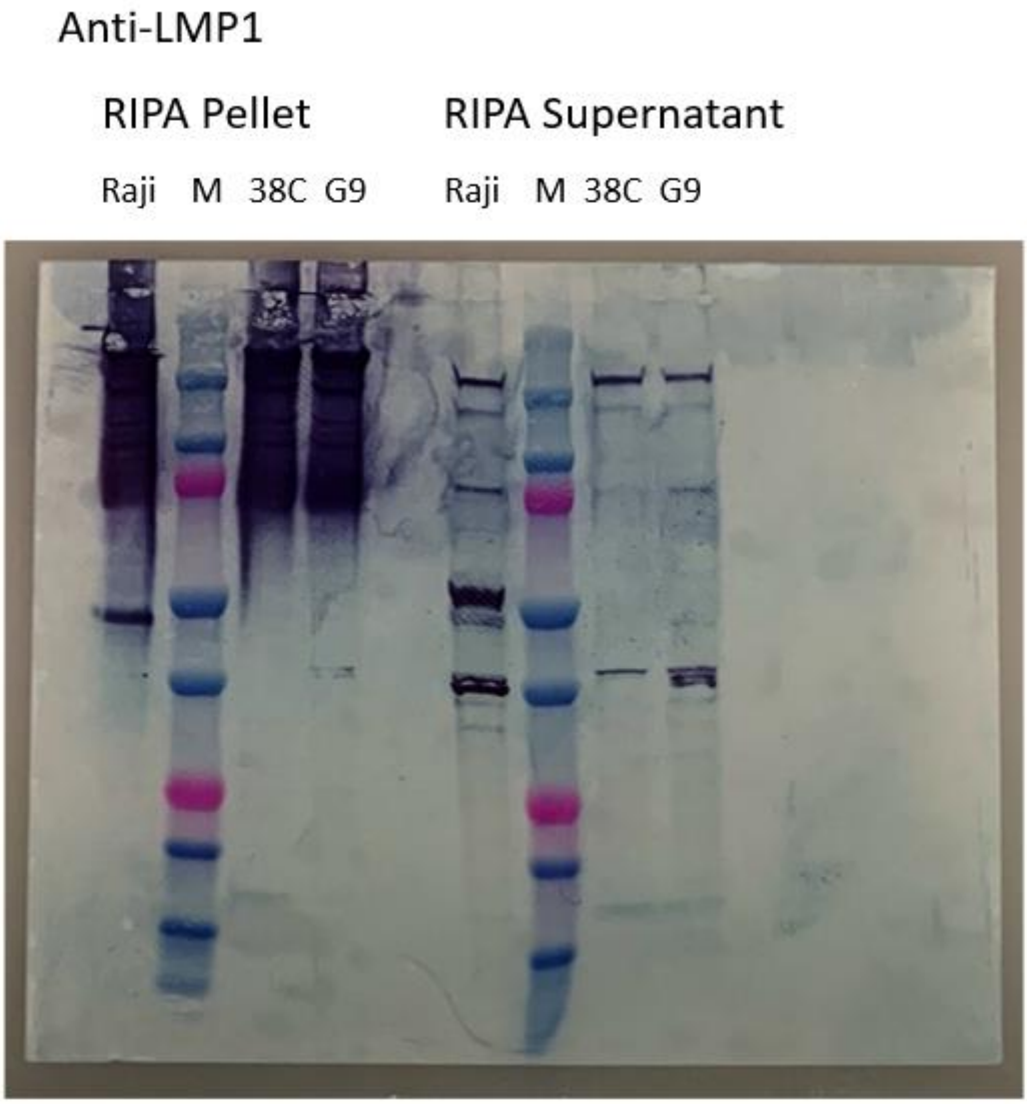


**Figure S2.** Original Western blot of LMP1 transformed 38C13 cells. 2 × $10^6$ cells were prepared using 200 μL of a RIPA buffer of Raji cells, 38C13 cells (38C) or 38C13-LMP1 clone G9, using a dual color BioRad protein standard (M). 40 μL of protein extracts was separated with SDS-PAGE, transferred to PDVF membranes, and then probed mouse anti-LMP1 monoclonal antibody (Millipore-Sigma MABF2248) at 1:1000 dilution, followed by a goat anti-mouse secondary (Southern Biotech), and then developed with a TMB membrane substrate (Surmodics).
